# The Effects of Tai Chi Intervention on Healthy Elderly by Means of Neuroimaging and EEG: A Systematic Review

**DOI:** 10.3389/fnagi.2018.00110

**Published:** 2018-04-18

**Authors:** Zhujun Pan, Xiwen Su, Qun Fang, Lijuan Hou, Younghan Lee, Chih C. Chen, John Lamberth, Mi-Lyang Kim

**Affiliations:** ^1^Department of Kinesiology, Mississippi State University, Starkville, MS, United States; ^2^Exercise Physiology Laboratory, College of Physical Education and Sports, Beijing Normal University, Beijing, China; ^3^Department of Sports, Leisure and Recreation, Soonchunhyang University, Asan, South Korea

**Keywords:** Tai Chi, aging, neuroimaging, EEG, neural plasticity

## Abstract

Aging is a process associated with a decline in cognitive and motor functions, which can be attributed to neurological changes in the brain. Tai Chi, a multimodal mind-body exercise, can be practiced by people across all ages. Previous research identified effects of Tai Chi practice on delaying cognitive and motor degeneration. Benefits in behavioral performance included improved fine and gross motor skills, postural control, muscle strength, and so forth. Neural plasticity remained in the aging brain implies that Tai Chi-associated benefits may not be limited to the behavioral level. Instead, neurological changes in the human brain play a significant role in corresponding to the behavioral improvement. However, previous studies mainly focused on the effects of behavioral performance, leaving neurological changes largely unknown. This systematic review summarized extant studies that used brain imaging techniques and EEG to examine the effects of Tai Chi on older adults. Eleven articles were eligible for the final review. Three neuroimaging techniques including fMRI (*N* = 6), EEG (*N* = 4), and MRI (*N* = 1), were employed for different study interests. Significant changes were reported on subjects' cortical thickness, functional connectivity and homogeneity of the brain, and executive network neural function after Tai Chi intervention. The findings suggested that Tai Chi intervention give rise to beneficial neurological changes in the human brain. Future research should develop valid and convincing study design by applying neuroimaging techniques to detect effects of Tai Chi intervention on the central nervous system of older adults. By integrating neuroimaging techniques into randomized controlled trials involved with Tai Chi intervention, researchers can extend the current research focus from behavioral domain to neurological level.

## Introduction

Older adults experience gradual regression of abilities. In addition to the physiological changes such as loss of muscular strength and declined vision, neurological ability declines with advanced aging. Tomasi and Volkow (2012) proposed that age-related decrease in motor and cognitive functions is associated with degeneration of the brain networks and changes in brain anatomy. Other studies indicated that decrease in functional connectivity as well as atrophy in gray matter and basal ganglia result in lack of motor control in older adults (Seidler et al., 2010; Hoffstaedter et al., 2015). However, aging process is reversible due to the plasticity and adaptivity of the human brain to experience-specific tasks (Adkins et al., 2006; Petzinger et al., 2010). Brain plasticity implies that reorganization of brain structure and functional connectivity is possible in older adults (Erickson et al., 2007). The finding suggested that appropriate intervention protocols such as exercise and motor training can counteract declines associated with advanced aging (Erickson et al., 2007; Seidler et al., 2010). For example, older adults participating in a 6-month aerobic exercise demonstrated better cardiovascular fitness and enhanced brain plasticity than the sedentary counterparts. Specifically, increased brain volume in gray and white matter were considered evidence of intact central nervous system and contributed to cognitive improvement (Colcombe et al., 2004, 2006). Bearing with the perception as to the significant role of brain plasticity in mitigating or even reversing the course of aging, researchers attempt to understand the neural mechanisms underlying exercise-related improvement in cognitive and motor performance.

Regular exercise is a practical approach to enhancing brain plasticity (Erickson et al., 2013; Voss et al., 2013). Tai Chi, a multimodal mind-body exercise integrating gracefulness, mindfulness, and gentleness, is a recommended form of physical activity for older adults (Wong et al., 2001). Benefits of practicing Tai Chi were reported in cognitive performance (Lam et al., 2011; Wayne et al., 2014) and motor functions such as postural control (Ni et al., 2014), fall prevention (Tousignant et al., 2013; Jain et al., 2017), muscle strength (Reid et al., 2016), and agility (Wayne et al., 2014). Given that neural plasticity shapes performance modification (Paré and Munoz, 2000), it is reasonable to assume that evolution of behavior associated with Tai Chi practice should be detected in the corresponding brain regions. Noninvasive neuroimaging techniques allow researchers to identify neural correlates of exercise-induced changes in the aging brain. Electroencephalography (EEG) produces spontaneous neuroelectric feedback on brain activity (Hatta et al., 2005; Fong et al., 2014). Magnetic Resonance Imaging (MRI) provides *in vivo* measures of brain anatomy and physiology (Giedd et al., 2015). Researchers used the technique to investigate structural changes in brain volume (Colcombe et al., 2006) and cortical thickness (Wei et al., 2013). Functional Magnetic Resonance Imaging (fMRI) detects brain connectivity based on blood oxygenation level-dependent (BOLD) signal in distinct brain regions (Fox et al., 2007). This technique has been applied to probe exercise-induced changes in brain activation and functional connection (Erickson et al., 2007; Seidler et al., 2010).

The current review summarized extant studies that applied Tai Chi to promote health for the following reasons. First, Tai Chi is an increasingly popular physical activity, which has been recommended for older adults and people with chronic disease. Second, despite the encouraging outcomes observed at the behavioral level, neural mechanisms underlying the promoted functions remain largely unknown (Voss et al., 2013). Neuroimaging (fMRI and MRI) and neuroelectric techniques (EEG) are the instruments that expand current knowledge on the correlates between neural plasticity and modified function. In this context, we aim to investigate three main issues: (1) Tai Chi-incurred benefits in older adults; (2) improved functions and corresponding changes in the brain; and (3) the direction of future study. To our knowledge, it is the first review to systematically investigate the benefits of Tai Chi exercise from the perspective of neural plasticity. With an increasing application of neuroimaging techniques, researchers should elevate the current study of interest from mere performance to neurological level.

## Methods

### Literature search

Five electronic databases (Google scholar, PubMed, Cochrane Library, Scopus, and Web of science) were searched for relevant studies published since 1990. The following terms were entered in multiple combinations, including older adults, elderly, seniors, aging, Tai Chi Chuan, Tai chi, Taichi, and Tai Ji. Terms for neuroimaging techniques include brain imaging, electroencephalography (EEG), event-related potentials (ERP) diffuse optical tomography (DOT), diffuse optical imaging (DOI), event-related optical signal (EROS), magnetic resonance imaging (MRI), Functional magnetic resonance imaging (fMRI), diffusion tensor imaging (DTI) arterial spin labeling (ASL), magnetoencephalography (MEG), computed tomography (CT), positron emission tomography (PET), and single-photon emission computerized tomography (SPECT). Manual search was conducted for known articles in the area by titles instead of keywords search.

### Eligibility criteria

Studies were eligible for inclusion if the following criteria were met: (1) subjects were healthy older adults or middle-aged adults (average age of Tai Chi group must be over 50); (2) Tai Chi was applied to exercise intervention; (3) brain imaging methods including MRI, fMRI, EEG, ERP etc. were used to assess variables of interest. The screening process consisted of two phases. First, two reviewers (XS & ZP) independently examined title, keywords, and abstracts of retrieved articles. In the second phase, a third author (QF) was responsible to deal with any disagreement between the reviewers.

Studies that failed to conform to one of the specified criteria were considered ineligible. To gain a comprehensive understanding of Tai Chi-related changes in the central nervous system of older adults, there were no restrictions on the types of studies. However, conference abstracts, review articles, monograph, and videos were excluded.

### Quality assessment

The methodological quality was assessed by Delphi list for quality assessment (Verhagen et al., 1998). To reduce the risk of bias in assessment, two reviewers (XS & ZP) independently scored the quality of the included articles. Inconsistencies between the two reviewers were solved after discussing with a third author.

### Data extraction

Study characteristics encompass basic information of the selected articles, including author(s) of study and year of publication, study design, place of study, sample size and attribution rate, intervention frequency and duration, age of subjects, and measures. Age of subjects refers to the average group age, which should be above 50. Measures applied to the studies must include neuroimaging (fMRI or MRI) or neuroelectric techniques (EEG). Rationale, findings, and practical implications were summarized according to the purpose, results, and conclusions of the retrieved studies.

## Results

### Study selection

**Figure 1:**
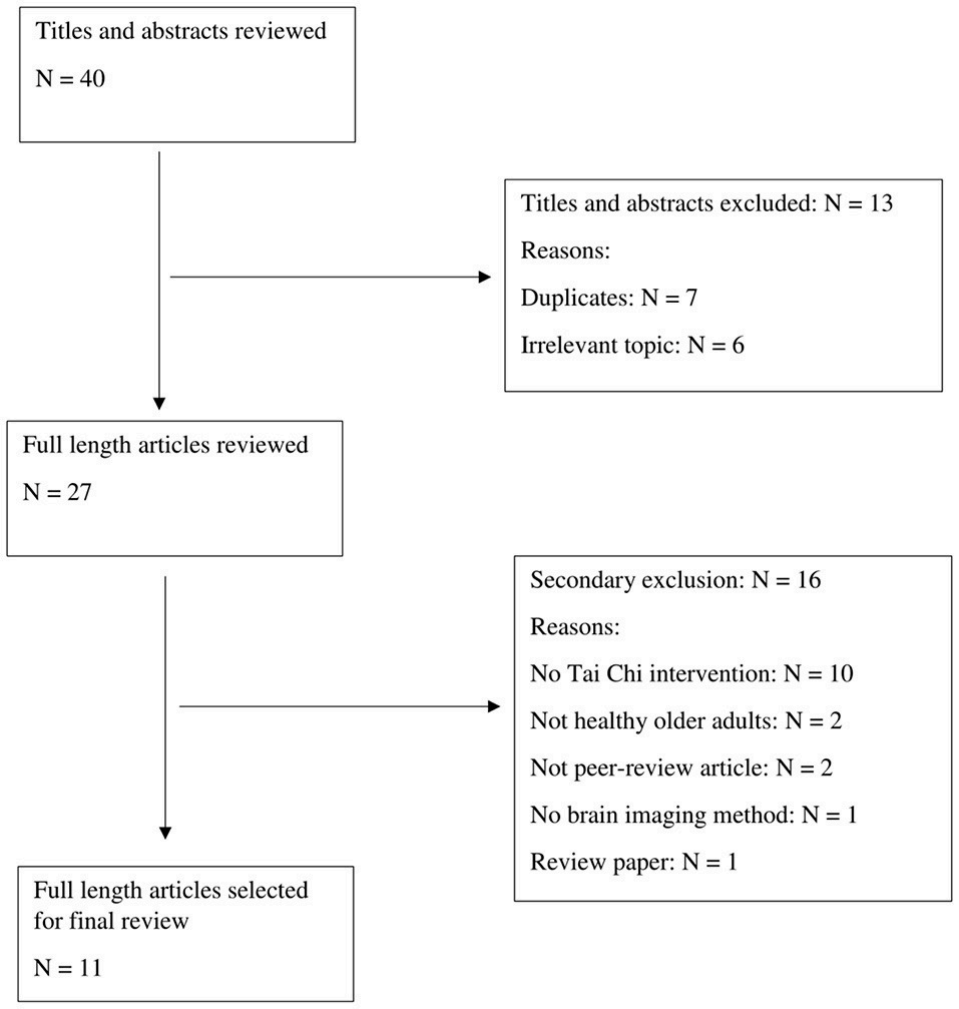
Article selection process.

A total of 40 articles were retrieved from the initial search. Examination of titles and abstracts excluded 13 irrelevant articles. Further analysis of the remaining 27 items screened off 16 articles for the following reasons: lack of Tai Chi intervention (*N* = 10), participants with health issues (*N* = 2), non-journal articles (*N* = 2), lack of brain imaging method (*N* = 1), and review paper (*N* = 1). Finally, 11 studies were eligible for full-text critical appraisal. Figure 1 indicates the study selection process.

### Study characteristics

Effects of Tai Chi intervention on participants' neurological changes received an increasing attention in recent years as nine of the included studies (*N* = 11) were published in the past 5 years. China is the major country where relevant studies were conducted (*N* = 8) due to the prevalence of Tai Chi in the region. Subjects were mostly seniors. The average age of Tai Chi group in the studies ranged between 50.5 and 68.6 years. The study design included pre- and post-tests (*N* = 1), RCT (*N* = 5), and Quasi-experiment (*N* = 5). Seven studies compared the subjects' performance of Tai Chi group with that of control group before and after the intervention. The other four studies examined the difference between experienced Tai Chi practitioners and people with a relatively sedentary lifestyle.

Scales and instruments such as Attention Network Test (ANT) and Memory Scale (MS) were used to assess behavioral and cognitive performance. On the other hand, MRI, fMRI, and EEG provided evidence of neural plasticity. MRI presented the image of brain structures (Wei et al., 2013, 2014; Zheng et al., 2015). fMRI examined functional connectivity (Li et al., 2014; Tao et al., 2016, 2017) and brain neural activity (Yin et al., 2014). EEG detected the spontaneous electric activity when a subject is performing a specific task (Liu et al., 2003; Field et al., 2010; Fong et al., 2014; Hawkes et al., 2014). Combining performance assessment with neuroimaging evidence allows researchers to investigate Tai Chi-induced outcomes at both behavioral and neural levels. Study characteristics are listed in Table 1.

**Table 1:** Summary of reviewed articles.

**References**	**Aim/Purpose**	**Design**	**Place of study**	**Sample size (attribution%)**	**Age group (year)**	**Intervention frequency & duration**	**Measurment instruments/Measures**	**Results**	**Conclusion**
Tao et al., 2017	To investigate the impact of Tai chi chuan and Baduanjin on the cognitive control network (CCN) especially on the DLPFC part. To explain the underlying mechanism of the fact that Taichi and Baduanjin can help improve mental control function.	RCT	Gulou District, Fuzhou City, China	TC: 21 BDJ:16 CG:25 *N* = 62	50–70 years TC: 62.38 ± 4.55 BDJ: 62.33 ± 3.88 CG: 59.76 ± 4.83	TC: 60 min/d, 5d/week, last for 12 weeks. BDJ: 60 min/d, 5d/ week, last for 12 weeks. CG: maintain original physical activity habits for 12 weeks.	WMS-CR fMRI	In TC, the rsFC decreases significantly between the DLPFC and the left SFG and ACC. In BDJ, the rsFC decreases significantly between the DLPFC and the left putamen and insula. Mental control improvement was negatively associated with rsFC DLPFC-putamen changes across all subjects.	The practice of Tai Chi and Baduanjin could significantly increases the participant's mental control function.
Tao et al., 2016	To investigate how longitudinal Tai Chi Chuan and Baduanjin can modulate memory function and HPC resting-state functional connectivity (rs-FC) in elderly adults and the relation between them.	RCT	Gulou District, Fuzhou City, China	TC: 21 BDJ:16 CG:25 *N* = 62	50–70 years TC: 62.38 ± 4.55 BDJ: 62.33 ± 3.88 CG: 59.76 ± 4.83	TC: 60min/d, 5d/week, last for 12 weeks. BDJ: 60min/d, 5d/week, last for 12 weeks. CG: maintain original physical activity habits for 12 weeks.	MQ (WMS-CR) fMRI	The MQ significantly increased in TC and BDJ compared to CG. The rs-FC between the bilateral HPC and mPFC significantly increased in the TC compared to the CG. The rs-FC increases between the bilateral HPC and mPFC were significantly associated with corresponding memory function improvement across all subjects.	Both Tai Chi Chuan and Baduanjin may be effective exercises to prevent memory decline during aging.
Field et al., 2010	To determine the immediate effects of a combined form of Tai chi/yoga.	pretest/posttest	Coral Gables, Florida, United States.	*N* = 38 *M* = 2.7 on the HSI (57% Caucasian, 14% Hispanic, 14% Asian, 5% Black, and 10% other.)	21–59 years (averaged 41.0)	20 min long, including 10min of Taichi movements and 10 min of yoga postures.	STAI EKG EEG Math computations	A trend for increased EEG theta activity was detected but the result was not significant enough (*p* = 0.10).	The increased relaxation may have contributed to the increased speed and accuracy noted on math computations following the Tai chi/yoga class.
Wei et al., 2013	To investigate whether brain structural difference existed between highly experienced TCC practitioners and healthy control non-TCC practitioners.	Quasi-experiment	Beijing, China	ETC: 22(7 males) CG: 18 *N* = 40	ETC: 52 ± 6 years CG: 54 ± 6 years	No intervention.	ANT (RT & error scores) MRI	ETC showed significantly thicker cortex in the right PG, IS and MFS, also in the left MOTS and LS. Thicker cortex in left MOTS and LS was associated with greater intensity of TCC practice.	TCC practice could induce regional structural change in the brain. TCC might share similar patterns of neural correlates with meditation and aerobic exercise.
Wei et al., 2014	To examine TCC-associated changes in the human brain's intrinsic architecture and the relevant gains in behavioral performance.	Quasi-experiment	Beijing, China	ETC: 22(7 males) CG: 18(8 males) *N* = 40	ETC: 52.4 ± 6.8 years CG: 54.8 ± 6.8 years	No intervention.	ANT R-fMRI 2dReHo	The ETC had significantly greater fHo in the right PosCG and less fHo in the left ACC and the right DLPFC. Increased functional homogeneity in the PosCG was correlated with TCC experience. Decreases in fHo in the left ACC and increases in fHo in the right PosCG both predicted performance gains on ANT.	These findings provide evidence for the functional plasticity of the brain's intrinsic architecture toward optimizing locally functional organization.
Fong et al., 2014	To determine the relationship between physical activity and the task-switching aspect of executive function. (by investigating the modulating roles of age, modality of physical activity, and type of cognitive function using behavioral and event-related potential (ERP) assessments.	Quasi-experiment	Taipei, Taiwan	OEE: 16 OTC: 16 OSL: 16 YA: 16 *N* = 64	OA: 65–75 years OEE: 68.37 ± 3.68 OTC:67.31 ± 4.92 OSL: 68.93 ± 4.28 YA: 20–30 years (22.43 ± 2.58)	No intervention	Questionnaire MMSE IPAQ ERP	YA, OEE, and OTC had significantly larger P3 amplitude compared with OSL under homogeneous and heterogeneous conditions, while no differences were observed among the former three groups. YA exhibited shorter P3 latency than OSL. The ERP findings support the model of the STAC.	Regular participation in endurance exercise and Tai Chi Chuan may have equivalent beneficial effects on cognition at the behavioral and neuroelectric levels. Age and participation in physical activity influence the relationship between physical activity and task-switching, and a positive relationship was observed regardless of the modality of physical activity and type of cognitive function.
Li et al., 2014	To investigate the functional plasticity in resting-state connectivity of the prefrontal cortex and MTL in older adults.	RCT	Beijing, China	IG: 17(9 men) CG: 17(11 men) *N* = 34	IG: 68.6 ± 5.7 years CG: 71.7 ± 4.0 years	Cognitive intervention (MT& EFT): 1-h session, 3 sessions/week, last for 6 weeks Tai Chi: 1-h session, 3 sessions/week, last for 6 weeks, Yang-Style 24-form Tai Chi Group counseling: 90-min session, 1 time/week, last for 6 weeks CG: two 120 min health-related lectures	Participants criteria: MoCA CES-D ADL standardized assessments: PALT digit span TMT Stroop Test CFT Health status: MOS SF-36 SSRS SWLS IWB Image acquisition: fMRI	After the training activities, IG showed dramatic increment in functional correlation between mPFC and PHC.L, and significantly increased functional connectivity between the mPFC and left PHG. CG showed significantly decreased connectivity between mPFC and MFG. IG showed significant correlation between the changes in the FC of mPFC-PHG and the changes in cognitive performance (CFT). IG's level of mPFC-PHC.L connectivity at the post-training scan correlated significantly with individual performance on the TMT.	Multimodal intervention could postpone the effects of aging and improve the function of the regions that are most heavily influenced by aging, as well as play an important role in preserving the brain and cognition during old age.
Yin et al., 2014	To examine the effects of a multimodal intervention on spontaneous brain activity in healthy older adults, and the relationship between individual differences in baseline spontaneous activity and intervention-induced changes in behavioral performance.	RCT	Beijing, China	IG: 17(9 men) CG: 17(11 men) *N* = 34	61–79 years IG: 68.6 ± 5.7 years CG: 71.7 ± 4.0 years	Cognitive intervention (MT& EFT): 1-h session, 3 sessions/week, last for 6 weeks Tai Chi: 1-h session, 3 sessions/week, last for 6 weeks, Yang-Style 24-form Tai Chi Group counseling: 90-min session, 1 time/week, last for 6 weeks CG: two 120 min health-related lectures	Participants criteria: MoCA CES-D ADL standardized assessments: PALT digit span TMT Stroop Test CFT Health status: MOS SF-36 SSRS SWLS IWB Image acquisition: fMRI	IG showed significantly increased ALFF in the right MFG, left SFG and left ACL, while the mean ALFF in all three ROIs was significantly reduced in CG. In IG, increased ALFF in the right MFG was significantly correlated with changes in the TMT and SWLS, and in the left ACL the ALFF increase was significantly correlated with changes in social support. The baseline ALFF in the right MFG was significantly correlated with changes in the TMT and SWLS.	Multimodal intervention is effective in improving cognitive functions and well-being and can induce functional changes in the aging brain. The study suggested resting-state ALFF as a marker of intervention-induced plasticity in older adults.
Zheng et al., 2015	To explore the regionally functional plasticity by using the ReHo method to do an exploratory analysis in the whole brain.	RCT	Beijing, China	IG: 17(9 men) CG: 17(11 men) *N* = 34	IG: 68.59 ± 5.65 years CG: 71.65 ± 4.00 years	Cognitive intervention (MT& EFT): 1-h session, 3 sessions/week, last for 6 weeks Tai Chi: 1-h session, 3 sessions/week, last for 6 weeks, Yang-Style 24-form Tai Chi Group counseling: 90-min session, 1 time/week, last for 6 weeks CG: two 120 min health-related lectures	Participants criteria: MoCA CES-D ADL standardized assessments: PALT digit span TMT Stroop Test CFT Health status: MOS SF-36 SSRS SWLS IWB Image acquisition: fMRI	In IG, ReHo significantly increased in the left STG and left PCL, but decreased in the left MTG. In CG, ReHo significantly decreased in the left STG and PCL and increased in bilateral MTG. In IG, the intervention-related ReHo changes in the left STG were significantly positively correlated with changes in the CFT, and changes in the right MTG were negatively correlated with increase in the total PALT scores.	The present study confirms that the combined intervention induces regionally brain functional reorganization, and it could optimize the intrinsic functional brain architecture in the temporal cortex and cerebellum in the normal elderly.
Hawkes et al., 2014	To determine if people who were long-term Tai Chi practitioners would show enhancements to executive function and aerobic capacity.	Quasi-experiment	Eugene and Springfield, Oregon	TC: 10(3 female) MEG: 16(6 female) AEG: 16(8 female) SG: 12(10 female) *N* = 54	TC: 55.4 ± 12.99 MEG: 48.63 ± 15.00 AEG: 44.09 ± 16.2 SG: 46.92 ± 12.81	No intervention	Rockport 1-mile walk VSTS test with EEG Button press response	TC and MEG showed significantly larger P3b switch amplitudes than sedentary controls, while AEG and SG did not differ significantly on this key executive function measure. P3b switch latency showed no significant differences between groups.	Long-term practice of Tai Chi may benefit a neurophysiological index of executive function.
Liu et al., 2003	To examine the different physiological and psychological effects of 24TJQ in middle-aged women.	Quasi-experiment	Osaka, Japan	SkG: 10 NG: 10 *N* = 20	SkG: 50.56 ± 5.45 NG: 53.66 ± 4.9	6-min 24TJQ exercise, with 3-min rest (before) and recovery (after)	Concurrently measure with the exercise: HR RR exercise intensity physical fitness test EEG (used telemeter system to avoid noise) EMG ST	SkG showed significantly higher values of alpha%-power in eye-closed rest and recovery period compared to NG, but they have nearly the same values during exercise. SG showed a tendency of higher beta%-power during experiment than NG. SG showed a significant increase of alpha%-power in central region compared to occipital region after exercise while there was no significant change in NG.	24TJQ is beneficial to keep or improve agility, flexibility, and muscle strength or endurance ability in middle-aged women, and gives a special effect on the cardio-respiratory system.

*RCT, randomized controlled trial; TC, Tai chi group; BDJ, Baduanjin group; CG: control group; WMS-CR, Wechsler Memory Scale–Chinese Revision; fMRI, functional magnetic resonance imaging; resting state functional connectivity, rsFC; DFPLC, bilateral dorsolateral prefrontal cortex; SFG, superior frontal gyrus; ACC, anterior cingulate cortex; MQ, memory quotient; HPC, hippocampus; mPFC, medial prefrontal cortex; HIS, Hollingshead Socioeconomic Index; STAI, the State Anxiety Inventory; HIS, Hollingshead Socioeconomic Index; STAI, State Anxiety Inventory; EKG, Electrocardiogram; EEG, Electroencephalogram; ETC, experienced Taichi practitioners; ANT, Attention Network Test; MRI, magnetic resonance imaging; PG, precentral gyrus; IS, insula sulcus; MFS, middle frontal sulcus; STG, superior temporal gyrus; MOTS, medial occipitotemporal sulcus; LS, lingual sulcus; R-fMRI, Resting-state functional magnetic resonance imaging; 2dReHo, 2d surface-based regional homogeneity; fHo, functional homogeneity; PosCG, post-central gyrus; ERP, event-related potential; OEE, older adults performing endurance exercise; OTC, older adults practicing Tai Chi Chuan; OSL, older adults with a sedentary lifestyle; YA, young adults; OA, older adults; MMSE, Mini-Mental State Examination; IPAQ, International Physical Activity Questionnaire; STAC, scaffolding theory of aging and cognition; MTL, medial temporal lobe; IG, Intervention group; MT, mnemonic training; EFT, executive function training; MoCA, Montreal Cognitive Assessment; CES-D, Center for Epidemiologic Depression Scale; ADL, activities of daily living; PALT, Paired Associative Learning Test; TMT, Trail Making Test; CFT, Category Fluency Test; MOS SF-36, Medical Outcomes Study Short Form-36; SSRS, Social Support Rating Scale; SWLS, Satisfaction with Life Scale; IWB, Index of Well-Being; mPFC, medial prefrontal cortex; HF, hippocampal formation; PHC, parahippocampal cortex; MFG, medial frontal gyrus; PHG, parahippocampal gyrus; FC, functional connectivity; SFG, superior frontal gyrus; ACL, anterior cerebellum lobe; ROI, region of interest; STG, superior temporal gyrus; PCL, posterior lobe of cerebellum; MTG, middle temporal gyrus; MEG, meditation plus exercise group; AEG, aerobic exercise group; SG, sedentary group; VSTS, Visuo-spatial task switch; 24TJQ, 24-style Taijiquan; SkG, skilled group; NG, novices' group; HR, heart rate; RR, respiratory rate; EMG, electromyography; ST, surface thermograph*.

### Quality assessment of eligible studies

Most of the included studies exhibited moderate (*N* = 5) to high (*N* = 5) quality of study design, with only one being categorized as low quality. Five cross-sectional studies aimed to identify different features between experienced Tai Chi practitioners and sedentary counterparts. Participants were recruited and allocated based on Tai Chi-related experiences and thus failed to meet the requirement of random allocation. For the studies without adopting intervention protocols, criterions such as similar at baseline (SB) and therapist blinded (TB) were not applicable to the studies (*N* = 5). Details of quality assessment are listed in Table 2.

**Table 2:** Quality assessment of reviewed studies.

**Study**	**EC**	**RA**	**CA**	**SAB**	**SB**	**TB**	**AB**	**DR**	**ITA**	**BC**	**PM**	**OSQ**
Tao et al., 2017	Yes	Yes	Yes	Yes	Yes	Yes	Yes	No	Yes	Yes	Yes	High
Tao et al., 2016	Yes	Yes	Yes	Yes	Yes	Yes	Yes	No	Yes	Yes	Yes	High
Field et al., 2010	No	CD	CD	CD	No	Yes	CD	Yes	Yes	NA	No	Low
Wei et al., 2013	Yes	No	No	NA	Yes	NA	Yes	Yes	NA	Yes	Yes	Moderate
Wei et al., 2014	Yes	No	No	NA	Yes	NA	Yes	Yes	NA	Yes	Yes	Moderate
Fong et al., 2014	Yes	No	No	NA	Yes	NA	Yes	Yes	NA	Yes	Yes	Moderate
Li et al., 2014	Yes	Yes	Yes	Yes	Yes	Yes	Yes	No	Yes	Yes	Yes	High
Yin et al., 2014	Yes	Yes	Yes	Yes	Yes	Yes	Yes	No	Yes	Yes	Yes	High
Zheng et al., 2015	Yes	Yes	Yes	Yes	Yes	Yes	Yes	No	Yes	Yes	Yes	High
Hawkes et al., 2014	Yes	No	No	NA	Yes	NA	Yes	Yes	NA	Yes	Yes	Moderate
Liu et al., 2003	Yes	No	No	NA	Yes	NA	Yes	Yes	NA	Yes	Yes	Moderate

*EC, eligibility criteria; RA, random allocation; CA, concealed allocation; SAB, similar at baseline; SB, subject blinded; TB, therapist blinded; AB, assessor blinded; DR, drop-out rate; ITA, intention-to-treat analysis; BC, between-group comparison; PM, points measures; OSQ, overall study quality; CD, cannot determine; NA, not applicable*.

### Summary of evidence

Summary of the studies involved with four categories of interest regarding the impacts of Tai Chi on brain structures, functional connectivity, neural activity, and electric activity. Details of the summarized evidence are displayed in Table 1.

### Brain structures

One study examined the differences in the brain structures between experienced Tai Chi practitioners and people lacking routine exercise. MRI image identified thicker cortex in the left and right hemisphere of long-term Tai Chi practitioners in comparison to the cortical regions of people with a sedentary lifestyle. The study suggested that cortex thickness in the left medial occipitotemporal sulcus and lingual sulcus is subject to the intensity of Tai Chi practice (Wei et al., 2013).

### Functional connectivity

Tai Chi-induced benefits in cognitive function were observed after elderly participants receiving a 6-week multimodal intervention, which consisted of Tai Chi exercise, group counseling, and cognitive training. Changes in functional connectivity included enhanced rsFC between the medial prefrontal cortex and the medial temporal lobe (Li et al., 2014). Given the fact that Tai Chi was the only form of physical activity in the intervention program, it is reasonable to assume that, to a certain extent, Tai Chi exercise contributed to the enhanced functional connectivity in correlation to improved cognitive performance.

Tao and colleagues examined correlates of mental control and functional connectivity (Tao et al., 2016, 2017). Participants who completed Tai Chi or a similar exercise (Baduanjin) over the 12-week intervention achieved a significant improvement in mental control and memory function. fMRI identified a significant decrease in the resting state functional connectivity (rsFC) between bilateral dorsolateral prefrontal cortex (DLPFC) and putamen, suggesting a negative relationship between mental control improvement and rsFC DLPFC-putamen connectivity (Tao et al., 2017). Superior memory function was found in alignment with increased rsFC between bilateral hippocampus and medial prefrontal cortex (Tao et al., 2016). Both studies substantiated the association between cognitive function and functional connectivity in prefrontal areas.

### Brain neural activity

Regional homogeneity (ReHo) and amplitude of low-frequency fluctuations (ALFF) in BOLD signal of fMRI revealed spontaneous neuronal activity (Zang et al., 2004; Fox and Raichle, 2007). The previous study found that ALFF declines with aging (Zuo and Xing, 2014). A multimodal intervention including Tai Chi, cognitive training, and group counseling benefited the intervention group in which strengthened ALFF in the middle frontal gyrus, superior frontal gyrus, and anterior cerebellum lobe was observed (Yin et al., 2014). Another study following similar protocols identified reorganized ReHo in the superior and middle temporal gyri, and the posterior lobe of the cerebellum (Zheng et al., 2015). Enhanced intrinsic brain activity is the evidence of Tai Chi-induced benefits in promoting cognitive functions.

### Brain electric activity

EEG detects brain electric activity, which is subject to physical activity. Participants showed better performance in math computation after Tai Chi and yoga practice (Field et al., 2010). Increased theta activity indicated immediate relaxation during exercise. The study suggested that Tai Chi and yoga exerted an immediate impact on brain activity. Brain plasticity was partially evident in that brain activity was adaptive to specific task.

Liu et al. (2003) investigated spontaneous brain activity of Tai Chi experts and novices during practice. Experts indicated a significantly higher alpha-wave amplitude than novices in eye-close resting and recovery period, suggesting that the experts could quickly and effectively reach a psychological relaxation. Also, the experts exhibited a higher beta-wave amplitude than novices, implying that experts tend to be more physiologically excited than novices throughout the practice. Experts indicated well-developed mind concentration capacity, which was evident in the alpha shift tendency from occipital lobe to central or frontal regions.

Cognitive function was assessed by event-related potential (ERP) while subjects conducting a task-switch test under homogeneous and heterogeneous conditions (Fong et al., 2014). P3 amplitude exhibited no difference between young adults and older adults with either regular endurance training or Tai Chi exercise. However, all three groups indicated significantly larger P3 amplitude than that indicated in the group of sedentary older adults. Similar P3 patterns between young and older adults participating in long-term exercise provided evidence regarding the benefits of endurance training and Tai Chi exercise on cognitive function. Another study examining P3b amplitude of subjects conducting task-switch test confirmed the benefits of long-term Tai Chi practice in the neural substrates of executive function (Hawkes et al., 2014).

## Discussion

The included studies reported positive outcomes of Tai Chi practice in older adults. Specifically, Tai Chi-induced benefits involved with superior capacities in respect to mental control (Tao et al., 2017), memory (Tao et al., 2016), fitness (Liu et al., 2003; Wei et al., 2013), cognition (Fong et al., 2014; Li et al., 2014; Wei et al., 2014; Yin et al., 2014), and executive function (Field et al., 2010; Hawkes et al., 2014; Zheng et al., 2015). Findings as to physiological and psychological improvement substantiated the significant role of Tai Chi practice in counteracting age-related decline in motor and cognitive function. More importantly, neural imaging techniques applied to the included studies provided evidence on the connection between improved performance and changes in the neural system. Aging brain still retains some plasticity, which may contribute to delaying or reversing neurological deterioration in the aging process (Kramer et al., 2004; Gabbard, 2011). Wei et al. (2013) identified effects of Tai Chi intervention on reshaping brain structures. The finding is consistent with previous studies, which observed greater cortical thickness in older adults after memory training (Engvig et al., 2010), meditation practice (Lazar et al., 2005), and aerobic exercise (Colcombe et al., 2006). Functional change is associated with the development of new neurons and synapses in the brain (Honey et al., 2009; Cai et al., 2014). In alignment with other forms of exercise, Tai Chi exercise mitigates brain structural and functional deficits (Seidler et al., 2010). Older adults maintaining an active lifestyle by routinely practicing Tai Chi indicated enhanced neural plasticity (Liu et al., 2003; Field et al., 2010; Fong et al., 2014; Hawkes et al., 2014). The included studies provided evidence-based explanation on the neural mechanisms underlying the exercise-induced improvement in motor and cognitive performance.

The reviewed studies only adopted tasks related to cognition, working memory, and executive function. Motor tasks, however, have yet been incorporated into EEG, fMRI, or MRI scan. In comparison to the EEG detection, which allows moderate physical activity, fMRI and MRI require subjects to maintain a resting state. Even small head motions may produce noise in brain scans (Power et al., 2012; Satterthwaite et al., 2012; Van Dijk et al., 2012), which proposed a challenge of integrate neuroimaging techniques into motor tasks. Researchers have designed a few tasks, which require a small range of motion such as finger tapping (Stoodley et al., 2012; Gardini et al., 2016), reaching and grasping (Culham et al., 2003), and lower limb joint motions (Kapreli et al., 2006). To expand knowledge on neural correlates of motor performance, feasible motor tasks should be developed to fit the setting of research employing the neuroimaging techniques.

Older adults experience reduced hemispheric asymmetry due to age-related deficits in neural connectivity (Cabeza, 2002). Evidence from fMRI scan indicated symmetric brain activations when older adults were performing cognitive tasks (Grady, 2000). A recent study involved with older adults also identified reduced asymmetry in movement patterns between dominant and non-dominant hand, suggesting a potential connection to the reduced hemispheric asymmetry (Przybyla et al., 2011). However, the theory remains to be an assumption without direct evidence from a study, which applies fMRI to motor tasks. By investigating the change in motor performance, whether it is associated with age-related degeneration or Tai Chi-incurred improvement, researchers can better understand neural mechanisms underlying the aging process.

The lack of robust empirical research on Tai Chi-incurred changes for older adults is a limitation of the review. The inherent risk of bias in the study design, paired with the limited literature, suggests the necessity of an increasing number of RCTs in this field. Only two of the included studies reported effect size, which makes it difficult to compare the effectiveness between studies. Future research should report the effect size so that critical conclusion can be reached based on statistical evidence.

## Conclusion

The literature review summarized 11 studies, which employed neuroimaging techniques and EEG to investigate effects of Tai Chi on hemispheric reorganization. The reviewed articles provide evidence that there may be cognitive improvement associated with modified brain activity, functional connectivity, and brain structures in older adults through Tai Chi exercise. Future studies should account for the potential connection between changed motor functions and corresponding neural mechanisms underlying the aging process. RCTs are needed to provide powerful evidence on the effect of Tai Chi intervention. In contrast to previous research focusing on performance, future studies should analyze the effects of intervention from the neurological standpoint. Applying neuroimaging techniques and EEG to Tai Chi intervention is worth investigating, which allows researchers to explore the neural mechanisms related to the effectiveness of Tai Chi exercise on counteracting the aging process.

## Author contributions

ZP and XS contributed to the conception and design of the review. ZP, XS, and QF applied the search strategy. All authors applied the selection criteria. All authors completed assessment of risk of bias. All authors analyzed the data and interpreted data. XS, QF, and ZP wrote this manuscript. LH, YL, CC, JL, and M-LK critically edited the manuscript.

### Conflict of interest statement

The authors declare that the research was conducted in the absence of any commercial or financial relationships that could be construed as a potential conflict of interest.

## References

[B1] Adkins D. L. Boychuk J. Remple M. S. Kleim J. A. (2006). Motor training induces experience-specific patterns of plasticity across motor cortex and spinal cord. J. Appl. Physiol. 101, 1776–1782. 10.1152/japplphysiol.00515.2006 16959909

[B2] Cabeza R. (2002). Hemispheric asymmetry reduction in older adults: the HAROLD model. Psychol. Aging 17, 85–100. 10.1037/0882-7974.17.1.85 11931290

[B3] Cai L. Chan J. S. Yan J. H. Peng K. (2014). Brain plasticity and motor practice in cognitive aging. Front. Aging Neurosci. 6:31. 10.3389/fnagi.2014.00031 24653695 PMC3947993

[B4] Colcombe S. J. Erickson K. I. Scalf P. E. Kim J. S. Prakash R. McAuley E.. (2006). Aerobic exercise training increases brain volume in aging humans. J. Gerontol. Ser. A Biol. Sci. Med. Sci. 61, 1166–1170. 10.1093/gerona/61.11.1166 17167157

[B5] Colcombe S. J. Kramer A. F. Erickson K. I. Scalf P. McAuley E. Cohen N. J.. (2004). Cardiovascular fitness, cortical plasticity, and aging. Proc. Natl. Acad. Sci. U.S.A. 101, 3316–3321. 10.1073/pnas.0400266101 14978288 PMC373255

[B6] Culham J. C. Danckert S. L. De Souza J. F. Gati J. S. Menon R. S. Goodale M. A. (2003). Visually guided grasping produces fMRI activation in dorsal but not ventral stream brain areas. Exp. Brain Res. 153, 180–189. 10.1007/s00221-003-1591-5 12961051

[B7] Engvig A. Fjell A. M. Westlye L. T. Moberget T. Sundseth Ø. Larsen V. A.. (2010). Effects of memory training on cortical thickness in the elderly. Neuroimage 52, 1667–1676. 10.1016/j.neuroimage.2010.05.041 20580844

[B8] Erickson K. I. Colcombe S. J. Wadhwa R. Bherer L. Peterson M. S. Scalf P. E.. (2007). Training-induced plasticity in older adults: effects of training on hemispheric asymmetry. Neurobiol. Aging 28, 272–283. 10.1016/j.neurobiolaging.2005.12.012 16480789

[B9] Erickson K. I. Gildengers A. G. Butters M. A. (2013). Physical activity and brain plasticity in late adulthood. Dialogues Clin. Neurosci. 15, 99–108. 23576893 10.31887/DCNS.2013.15.1/kerickson PMC3622473

[B10] Field T. Diego M. Hernandez-Reif M. (2010). Tai chi/yoga effects on anxiety, heartrate, EEG and math computations. Complement. Ther. Clin. Pract. 16, 235–238. 10.1016/j.ctcp.2010.05.014 20920810 PMC2950830

[B11] Fong D. Y. Chi L. K. Li F. Chang Y. K. (2014). The benefits of endurance exercise and Tai Chi Chuan for the task-switching aspect of executive function in older adults: an ERP study. Front. Aging Neurosci. 6:295. 10.3389/fnagi.2014.00295 25389403 PMC4211410

[B12] Fox M. D. Raichle M. E. (2007). Spontaneous fluctuations in brain activity observed with functional magnetic resonance imaging. Nat. Rev. Neurosci. 8, 700–711. 10.1038/nrn2201 17704812

[B13] Fox M. D. Snyder A. Z. Vincent J. L.. (2007). Intrinsic fluctuations within cortical systems account for intertrial variability in human behavior. Neuron 56, 171–184. 10.1016/j.neuron.2007.08.023 17920023

[B14] Gabbard C. P. (2011). Lifelong Motor Development. San Francisco, CA: Pearson Higher Ed.

[B15] Gardini S. Venneri A. McGeown W. J. Toraci C. Nocetti L. Porro C. A.. (2016). Brain activation patterns characterizing different phases of motor action: execution, choice and ideation. Brain Topogr. 29, 679–692. 10.1007/s10548-016-0491-5 27072014

[B16] Giedd J. N. Raznahan A. Alexander-Bloch A. Schmitt E. Gogtay N. Rapoport J. L. (2015). Child psychiatry branch of the National Institute of Mental Health longitudinal structural magnetic resonance imaging study of human brain development. Neuropsychopharmacology 40, 43–49. 10.1038/npp.2014.236 25195638 PMC4262916

[B17] Grady C. L. (2000). Functional brain imaging and age-related changes in cognition. Biol. Psychol. 54, 259–281. 10.1016/S0301-0511(00)00059-4 11035226

[B18] Hatta A. Nishihira Y. Kim S. R. Kaneda T. Kida T. Kamijo K.. (2005). Effects of habitual moderate exercise on response processing and cognitive processing in older adults. Jpn. J. Physiol. 55, 29–36. 10.2170/jjphysiol.R2068 15796787

[B19] Hawkes T. D. Manselle W. Woollacott M. H. (2014). Tai Chi and meditation-plus-exercise benefit neural substrates of executive function: a cross-sectional, controlled study. J. Complement. Integr. Med. 11, 279–288. 10.1515/jcim-2013-0031 25294719

[B20] Hoffstaedter F. Grefkes C. Roski C. Caspers S. Zilles K. Eickhoff S. B. (2015). Age-related decrease of functional connectivity additional to gray matter atrophy in a network for movement initiation. Brain Struct. Funct. 220, 999–1012. 10.1007/s00429-013-0696-2 24399178 PMC7994298

[B21] Honey C. J. Sporns O. Cammoun L. Gigandet X. Thiran J. P. Meuli R.. (2009). Predicting human resting-state functional connectivity from structural connectivity. Proc. Natl. Acad. Sci. U.S.A. 106, 2035–2040. 10.1073/pnas.0811168106 19188601 PMC2634800

[B22] Jain A. Taylor J. Sanzo P. Zerpa C. (2017). The effect of Tai Chi on functional lower extremity mobility and strength, ankle proprioception, and postural adaptation in older adults. American J. Med. Med. Sci. 7, 229–237. 10.5923/j.ajmms.20170706.01

[B23] Kapreli E. Athanasopoulos S. Papathanasiou M. Van Hecke P. Strimpakos N. Gouliamos A.. (2006). Lateralization of brain activity during lower limb joints movement. An fMRI study. Neuroimage 32, 1709–1721. 10.1016/j.neuroimage.2006.05.043 16859927

[B24] Kramer A. F. Bherer L. Colcombe S. J. Dong W. Greenough W. T. (2004). Environmental influences on cognitive and brain plasticity during aging. J. Gerontol. Ser. A Biol. Sci. Med. Sci. 59, M940–M957. 10.1093/gerona/59.9.M940 15472160

[B25] Lam L. C. Chau R. C. Wong B. M. Fung A. W. Lui V. W. Tam C. C.. (2011). Interim follow-up of a randomized controlled trial comparing Chinese style mind body (Tai Chi) and stretching exercises on cognitive function in subjects at risk of progressive cognitive decline. Int. J. Geriatr. Psychiatry 26, 733–740. 10.1002/gps.2602 21495078

[B26] Lazar S. W. Kerr C. E. Wasserman R. H.. (2005). Meditation experience is associated with increased cortical thickness. Neuroreport 16:1893. 10.1097/01.wnr.0000186598.66243.19 16272874 PMC1361002

[B27] Li R. Zhu X. Yin S. Niu Y. Zheng Z. Huang X.. (2014). Multimodal intervention in older adults improves resting-state functional connectivity between the medial prefrontal cortex and medial temporal lobe. Front. Aging Neurosci. 6:39. 10.3389/fnagi.2014.00039 24653698 PMC3948107

[B28] Liu Y. Mimura K. Wang L. Ikuda K. (2003). Physiological benefits of 24-style Taijiquan exercise in middle-aged women. J. Physiol. Anthropol. Appl. Human Sci. 22, 219–225. 10.2114/jpa.22.219 14519910

[B29] Ni M. Mooney K. Richards L. Balachandran A. Sun M. Harriell K.. (2014). Comparative impacts of tai chi, balance training, and a specially-designed yoga program on balance in older fallers. Arch. Phys. Med. Rehabil. 95, 1620.e30–1628.e30. 10.1016/j.apmr.2014.04.022 24835753

[B30] Paré M. Munoz D. P. (2000). Immediate neural plasticity shapes motor performance. J. Neurosci. 20:RC52. 10.1523/JNEUROSCI.20-01-j0005.2000 10627629 PMC6774144

[B31] Petzinger G. M. Fisher B. E. Van Leeuwen J. E. Vukovic M. Akopian G. Meshul C. K.. (2010). Enhancing neuroplasticity in the basal ganglia: the role of exercise in Parkinson's disease. Mov. Disord. 25(Suppl. 1), S141–S145. 10.1002/mds.22782 20187247 PMC4111643

[B32] Power J. D. Barnes K. A. Snyder A. Z. Schlaggar B. L. Petersen S. E. (2012). Spurious but systematic correlations in functional connectivity MRI networks arise from subject motion. Neuroimage 59, 2142–2154. 10.1016/j.neuroimage.2011.10.018 22019881 PMC3254728

[B33] Przybyla A. Haaland K. Y. Bagesteiro L. B.. (2011). Motor asymmetry reduction in older adults. Neurosci. Lett. 489, 99–104. 10.1016/j.neulet.2010.11.074 21144883 PMC3422634

[B34] Reid K. Price L. Harvey W. (2016). Changes in leg muscle strength and power after Tai Chi exercise in patients with symptomatic knee osteoarthritis. Osteoarthr. Cartil. 24:S430 10.1016/j.joca.2016.01.776

[B35] Satterthwaite T. D. Wolf D. H. Loughead J. Ruparel K. Elliott M. A. Hakonarson H.. (2012). Impact of in-scanner head motion on multiple measures of functional connectivity: relevance for studies of neurodevelopment in youth. Neuroimage 60, 623–632. 10.1016/j.neuroimage.2011.12.063 22233733 PMC3746318

[B36] Seidler R. D. Bernard J. A. Burutolu T. B. Fling B. W. Gordon M. T. Gwin J. T.. (2010). Motor control and aging: links to age-related brain structural, functional, and biochemical effects. Neurosci. Biobehav. Rev. 34, 721–733. 10.1016/j.neubiorev.2009.10.005 19850077 PMC2838968

[B37] Stoodley C. J. Valera E. M. Schmahmann J. D. (2012). Functional topography of the cerebellum for motor and cognitive tasks: an fMRI study. Neuroimage 59, 1560–1570. 10.1016/j.neuroimage.2011.08.065 21907811 PMC3230671

[B38] Tao J. Chen X. Egorova N. Liu J. Xue X. Wang Q.. (2017). Tai Chi Chuan and Baduanjin practice modulates functional connectivity of the cognitive control network in older adults. Sci. Rep. 7:41581. 10.1038/srep41581 28169310 PMC5294576

[B39] Tao J. Liu J. Egorova N. Chen X. Sun S. Xue X.. (2016). Increased hippocampus–medial prefrontal cortex resting-state functional connectivity and memory function after tai chi chuan practice in elder adults. Front. Aging Neurosci. 8:25. 10.3389/fnagi.2016.00025 26909038 PMC4754402

[B40] Tomasi D. Volkow N. D. (2012). Aging and functional brain networks. Mol. Psychiatry 17, 471, 549–558. 10.1038/mp.2012.27 PMC3193908 21727896

[B41] Tousignant M. Corriveau H. Roy P. M.. (2013). Efficacy of supervised Tai Chi exercises versus conventional physical therapy exercises in fall prevention for frail older adults: a randomized controlled trial. Disabil. Rehabil. 35, 1429–1435. 10.3109/09638288.2012.737084 23167499

[B42] Van Dijk K. R. Sabuncu M. R. Buckner R. L. (2012). The influence of head motion on intrinsic functional connectivity MRI. Neuroimage 59, 431–438. 10.1016/j.neuroimage.2011.07.044 21810475 PMC3683830

[B43] Verhagen A. P. de Vet H. C. de Bie R. A. Kessels A. G. Boers M. Bouter L. M.. (1998). The Delphi list: a criteria list for quality assessment of randomized clinical trials for conducting systematic reviews developed by Delphi consensus. J. Clin. Epidemiol. 51, 1235–1241. 10.1016/S0895-4356(98)00131-0 10086815

[B44] Voss M. W. Erickson K. I. Prakash R. S. Chaddock L. Kim J. S. Alves H.. (2013). Neurobiological markers of exercise-related brain plasticity in older adults. Brain Behav. Immun. 28, 90–99. 10.1016/j.bbi.2012.10.021 23123199 PMC3544982

[B45] Wayne P. M. Walsh J. N. Taylor-Piliae R. E. Wells R. E. Papp K. V. Donovan N. J.. (2014). Effect of Tai Chi on cognitive performance in older adults: systematic review and meta-analysis. J. Am. Geriatr. Soc. 62, 25–39. 10.1111/jgs.12611 24383523 PMC4055508

[B46] Wei G. X. Dong H. M. Yang Z. Luo J. Zuo X. N. (2014). Tai Chi Chuan optimizes the functional organization of the intrinsic human brain architecture in older adults. Front. Aging Neurosci. 6:74. 10.3389/fnagi.2014.00074 24860494 PMC4029006

[B47] Wei G.-X. Xu T. Fan F.-M.. (2013). Can Taichi reshape the brain? A brain morphometry study. PLoS ONE 8:e61038. 10.1371/journal.pone.0061038 23585869 PMC3621760

[B48] Wong A. M. Lin Y. C. Chou S. W. Tang F. T. Wong P. Y. (2001). Coordination exercise and postural stability in elderly people: effect of Tai Chi Chuan. Arch. Phys. Med. Rehabil. 82, 608–612. 10.1053/apmr.2001.22615 11346836

[B49] Yin S. Zhu X. Li R. Niu Y. Wang B. Zheng Z.. (2014). Intervention-induced enhancement in intrinsic brain activity in healthy older adults. Sci. Rep. 4:7309. 10.1038/srep07309 25472002 PMC4255189

[B50] Zang Y. Jiang T. Lu Y. He Y. Tian L. (2004). Regional homogeneity approach to fMRI data analysis. Neuroimage 22, 394–400. 10.1016/j.neuroimage.2003.12.030 15110032

[B51] Zheng Z. Zhu X. Yin S. Wang B. Niu Y. Huang X.. (2015). Combined cognitive-psychological-physical intervention induces reorganization of intrinsic functional brain architecture in older adults. Neural Plast. 2015:713104. 10.1155/2015/713104 25810927 PMC4355335

[B52] Zuo X. N. Xing X. X. (2014). Test-retest reliabilities of resting-state FMRI measurements in human brain functional connectomics: a systems neuroscience perspective. Neurosci. Biobehav. Rev. 45, 100–118. 10.1016/j.neubiorev.2014.05.009 24875392

